# Anatomy driven optimization strategy for total marrow irradiation with a volumetric modulated arc therapy technique

**DOI:** 10.1120/jacmp.v13i1.3653

**Published:** 2012-01-05

**Authors:** Pietro Mancosu, Pierina Navarria, Luca Castagna, Antonella Roggio, Chiara Pellegrini, Giacomo Reggiori, Antonella Fogliata, Francesca Lobefalo, Simona Castiglioni, Filippo Alongi, Luca Cozzi, Armando Santoro, Marta Scorsetti

**Affiliations:** ^1^ Radiation Oncology Dept. Humanitas Cancer Center Milano (Rozzano) Italy; ^2^ Bone Marrow Transplantation Unit Humanitas Cancer Center Milano (Rozzano) Italy; ^3^ Medical Physics Unit Oncology Institute of Southern Switzerland Bellinzona Switzerland; ^4^ Department of Medical Oncology and Hematology Humanitas Cancer Center Milano (Rozzano) Italy

**Keywords:** RapidArc, total marrow irradiation, volumetric‐modulated arc therapy (VMAT), planning optimization

## Abstract

The purpose of this study was to evaluate the possibility of dose distribution optimization for total marrow irradiation (TMI) employing volumetric‐modulated arc therapy (VMAT) with RapidArc (RA) technology setting isocenter's positions and jaw's apertures according to patient's anatomical features. Plans for five patients were generated with the RA engine (PROIII): eight arcs were distributed along four isocenters and simultaneously optimized with collimator set to 90°. Two models were investigated for geometrical settings of arcs: (1) in the “symmetric” model, isocenters were equispaced and field apertures were set the same for all arcs to uniformly cover the entire target length; (2) in the “anatomy driven” model, both field sizes and isocenter positions were optimized in order to minimize the target volume near the field edges (i.e., to maximize the freedom of motion of MLC leaves inside the field aperture (for example, avoiding arcs with ribs and iliac wings in the same BEV)). All body bones from the cranium to mid of the femurs were defined as PTV; the maximum length achieved in this study was 130 cm. Twelve (12) Gy in 2 Gy/fractions were prescribed in order to obtain the covering of 85% of the PTV by 100% of the prescribed dose. For all organs at risk (including brain, optical structures, oral and neck structures, lungs, heart, liver, kidneys, spleen, bowels, bladder, rectum, genitals), planning strategy aimed to maximize sparing according to ALARA principles, looking to reach a mean dose lower than 6 Gy (i.e., 50% of the prescribed dose). Mean MU/fraction resulted 3184±354 and 2939±264 for the two strategies, corresponding to a reduction of 7% (range −2% to 13%) for (1) and (2). Target homogeneity, defined as $D_{2\%}-D_{98\%}$ was 18% better for (2). Mean dose to the healthy tissue, defined as body minus PTV, had 10% better reduction with (2). The isocenter's position and the jaw's apertures are significant parameters in the optimization of the TMI with RA technique, giving the medical physicist a crucial role in driving the optimization and thus obtaining the best plan. A clinical protocol started in our department in October 2010.

PACS numbers: 87.55.de, 87.55.dk, 87.56.nk, 87.57.uq

## I. INTRODUCTION

Total marrow irradiation (TMI) is a new potential approach for conditioning regimen in patients scheduled for hematopoietic cell transplantation in multiple myeloma, leukemia, and lymphomas. The aim of this innovative technique is to improve the coverage of target hematopoietic or lymphoid tissues while reducing the involvement of the remaining healthy tissues in the body, and thus reducing toxicities with respect to the standard total body irradiation (TBI) where all the body is irradiated homogenously.

Wilkie et al.^(^ ^1^ ^)^ reported a feasibility study for TMI with conventional linacs and application of intensity‐modulated fields in a multiple isocenter setting. The study showed, on an anthropomorphic phantom, that intensity‐modulated techniques might reduce the dose to organs at risk (OAR) by 29%–65% compared to conventional total body irradiation. A similar approach was followed by Aydogan et al.^(^ ^2^ ^)^ Other analogue investigations were performed by means of helical tomotherapy (HT)‐based approaches.^(^ ^3^ ^,^ ^4^ ^)^ The City of Hope group in Duarte (USA) reported on both simulation studies and treatment of 21 patients.^(^ ^4^ ^)^ For a group of 13 patients treated for multiple myeloma on a dose escalation protocol (from 10 to 16 Gy at 2 Gy daily/twice daily), the authors demonstrated that median organ doses were 15%–65% of that received from the gross target volume. In terms of toxicity, primarily grade 1–2 acute effects were observed, and no patient showed grade 4 toxicity.

In previous works at our institution,^(^ ^5^ ^)^ at University of Chicago^(^ ^6^ ^)^ and at City of Hope National Medical Center,^(^ ^7^ ^)^ the possibility to use volumetric‐modulated arc therapy (VMAT) by RapidArc, for the generation of clinically acceptable TMI plans, was demonstrated. RapidArc (Varian, Palo Alto, California, USA) is a VMAT technique based on the simultaneous optimization of multileaf collimator (MLC) shape, field modulation, and gantry rotation speed.^(^ ^8^ ^)^

In particular, in our previous work, jaw apertures were set symmetrically; the isocenter positions were regularly set maintaining the same distance between one isocenter and those following, in order to cover the whole body.

In this work we have evaluated the possibility to further optimize the RapidArc dose distribution of TMI by setting the isocenter and jaw apertures according to the patient's anatomy. Our assumption is that this helps the optimizer in avoiding conflicts and, hence, allows better dose distribution and a greater sparing of healthy tissues.

## II. MATERIALS AND METHODS

### A.1 Data acquisition

A retrospective analysis was performed on five patients. Computed tomography (CT) datasets from the top of the skull to the knees were acquired with a 3 mm slice thickness from a 16‐slice CT system. Patients were simulated free‐breather in a supine position with the arms immobilized at their sides.

The planning target volume (PTV) was defined, in agreement with our institute's hematologists and with the literature, as being all the skeletal bones with exclusion of the mandible and maxillary structures, the hands, and the lower legs from mid‐femurs.^(^ ^4^ ^)^ The mean PTV volume was 7.4 l, ranging 5.8–10.8 l. OAR included in the study were: eyes, lenses, parotids, oral cavity, thyroid, trachea (including also the esophagus), lungs, heart, stomach, kidneys, liver, spleen, bowel cavity, bladder, rectum. and genitals. A healthy tissue, defined as Body‐PTV with a further crop of 1.5 cm, was defined in order to better conform the dose.

Dose prescription to the PTV was set to 12 Gy in six fractions (i.e., 2Gy/fraction), twice daily. Dose was normalized so that 100% of the prescription dose covered 85% of the PTV (i.e., $V_{100\%}=85\%$). Planning objectives for PTV aimed to limit minimum and maximum doses. No specific dose‐volume planning objectives were defined for the organ at risks (OARs). Plans were optimized so as to maximize the sparing of each OAR in order to reduce median doses ($D_{50}$) below 6 Gy (i.e., less than 50% of the prescription dose).^(^ ^4^ ^)^

### A.2 Plan optimization

The TMI treatment plans in the present study were prepared using the RapidArc provided within the Eclipse treatment planning system, version 10.0 (Varian Medical Systems, Palo Alto, CA) on a standalone Dell Precision T5400 workstation personal computer with 8‐way 2.5 GHz intel Pentium III and 20478 MB of RAM. The progressive resolution optimizer algorithm PROIII (Varian Medical Systems, Palo Alto, CA), version 10.0, was used to optimize all RapidArc plans. This version allows the simultaneous optimization of a maximum of 10 full arcs and operates with a 64‐bit client without memory restrictions that are typical of 32‐bit applications. Final dose calculation was performed using a preclinical version of the Acuros photon dose algorithm (Transpire, Inc., Gig Harbor, WA) using a grid of 2.5 mm.^(^ ^9^ ^,^ ^10^ ^)^ The average time for optimization was around 1 hour and 1.5 hours for dose calculation.

**Figure 1:**
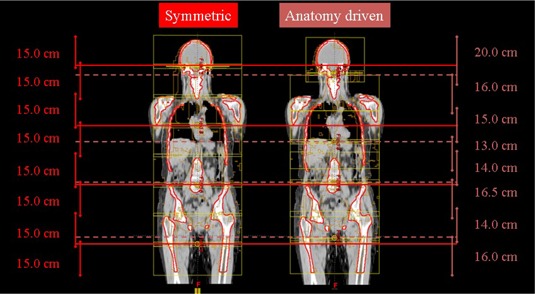
Overview of the different isocenter and jaw settings in the two different approaches.

For each patient, two plans with different isocenter and jaw settings were generated. Eight 6 MV coplanar arcs (360°) were optimized simultaneously. The collimator angle is discussed in the next paragraph. The eight arcs had a total number of four isocenters (two adjacent arcs had the same isocenter) using asymmetric jaw settings to cover the entire PTV length. Field width was set to 40 cm, while field length ranged from 15 to 16 cm. Each arc overlapped with the previous and following ones for at least 2 cm on each side to eliminate any need of matching planes. With an overlapping region of some centimeters, the differences in delivered dose distributions with respect to planning, due to small patient misalignment between isocenters, are minimized.^(^ ^11^ ^)^ For each arc, the collimator rotation was set to 90°; thus, the MLC was parallel to the beam eye view frame (i.e., parallel to the cranial–caudal direction). Two models were investigated for the geometrical setting of arcs: (1) in the “symmetric” model, isocenters were equispaced and field apertures were set identical for all arcs to uniformly cover the entire target length; (2) in the “anatomy driven” model, both field sizes and isocenter positions were decided by taking into consideration the specific anatomy of the patient. In particular, these parameters were optimized in order to minimize the target volume near the field edges (i.e., to maximize the freedom of motion of MLC leaves inside the field aperture in order to, for example, avoid arcs with ribs and iliac wings in the same BEV) (Fig. 1).

All the plans were optimized starting from a template of objectives based on our previous study^(^ ^5^ ^)^ adjusting, when necessary, the dose‐volume constraints during the optimization in order to properly cover the target and spare the organs at risk. The entire gantry rotation was described in the optimization process by a sequence of 177 control points (i.e., one every 2°). Plans for RapidArc were optimized by selecting a maximum dose rate of 600 MU/min.

### A.3 Data analysis

The distance between each couple of subsequent isocenters was measured and the standard deviation of these measurements was calculated (SDID) for both approaches. The same procedure was performed for the jaw apertures (SDJA). Plans were quantitatively evaluated from dose‐volume histograms (DVH) analysis, assessing for PTV: mean dose, percentage of volume receiving 110% of the prescription dose $V_{110\%}$, and target homogeneity index (HI) defined as $D_{2\%}–D_{98}\%$; for OARs, the analysis included the median dose ($D_{50\%}$) and $D_{10\%}$, and a t‐student test was performed to assess the statistical significance of the results. Delivery parameters were recorded in terms of MU per fraction and effective beam on time (BOT), which was defined as the pure beam on time plus the time needed to reset the system between beams without any additional dead time due to external reasons.

## III. RESULTS

**Figure 2:**
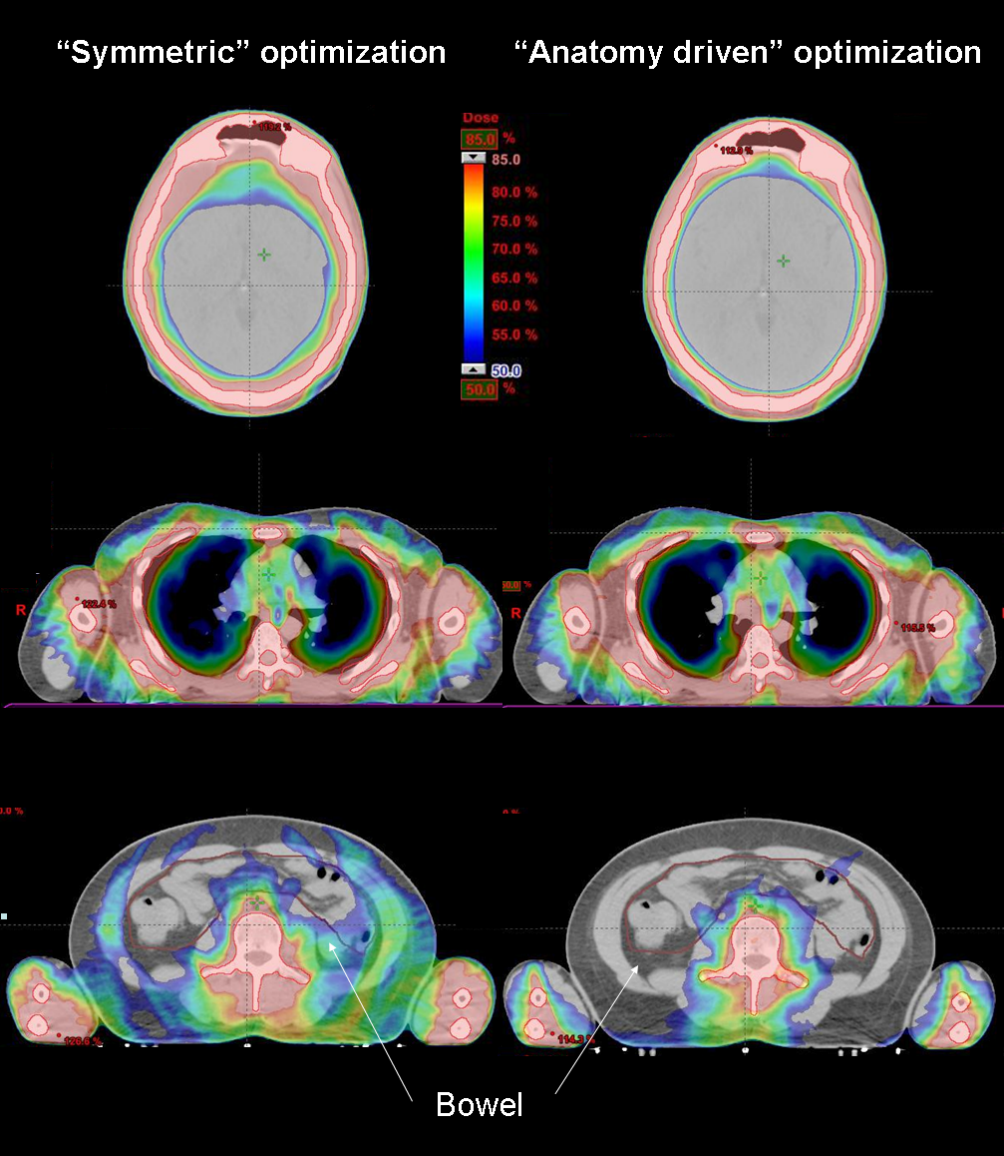
Transversal view of three slices belonging to different anatomical districts: head, thorax, and abdomen. The different dose distribution obtained with the two different methods are shown. The differences occur especially where OARs with big volumes are considered.

**Figure 3:**
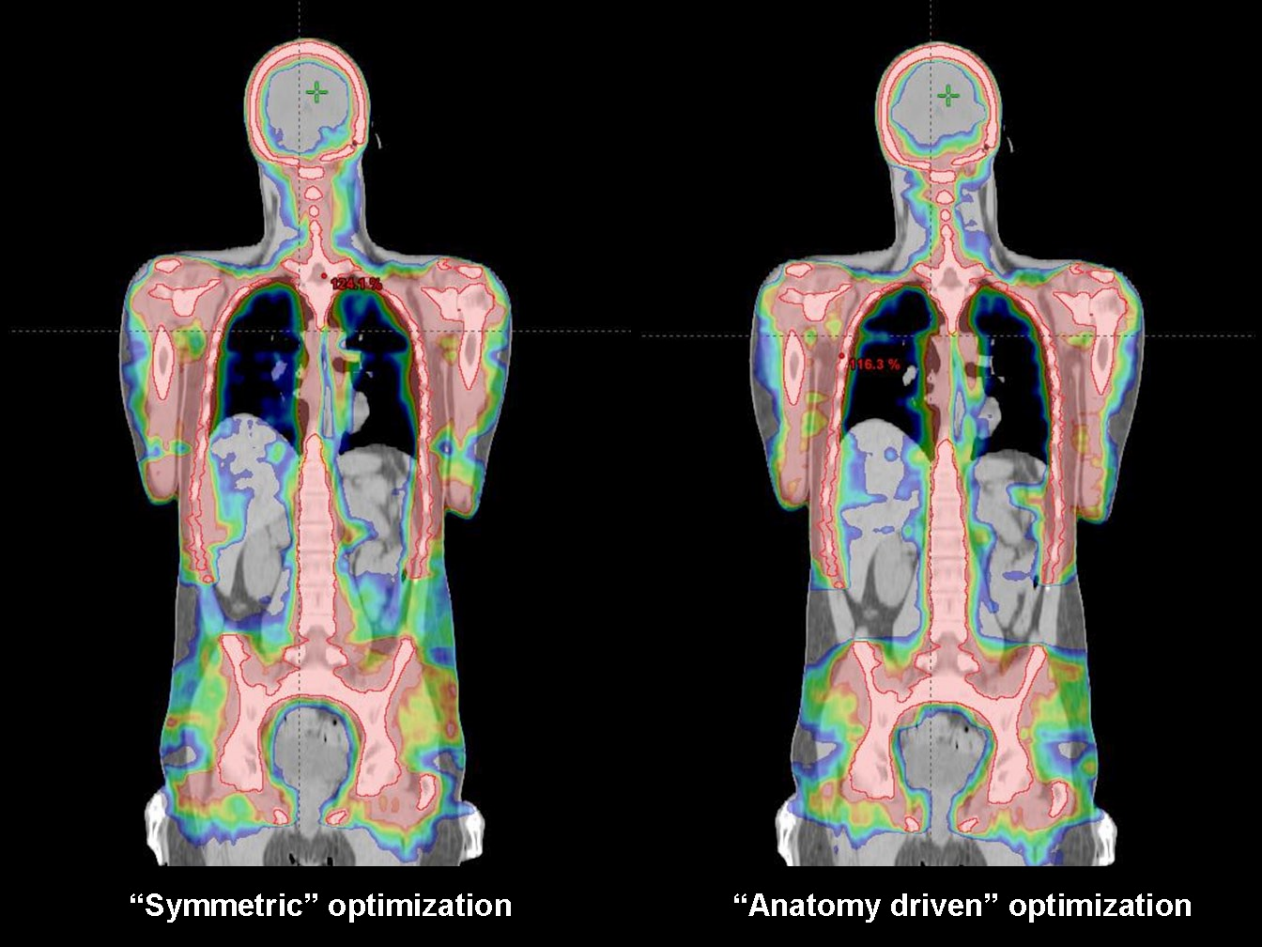
Whole body frontal view of the two different plans. The different dose distribution can be observed, particularly evident in the abdominal region (i.e., bowel).

By definition, SDID and SDJA for the model (1) were equal to zero, while for the model (2), mean SDID of 17 mm and SDJA of 32 mm were found. In Figs. 2 and 3, dose distributions for one patient for axial and coronal views are shown, providing a qualitative overview. A colorwash scheme ranging from 6.0 Gy to 10.2 Gy (i.e., 50%–85% of the prescribed dose) was used to demonstrate target coverage and dose sparing at all organs at risk. Similar results were obtained for the other patients.

Mean MU/fraction resulted 3184±354 and 2939±264 for the two strategies, equivalent to a reduction of 7% (range −2% to 13%) for the anatomy driven optimization, corresponding to an average of 398±67 and 367±62 MU per arc. The total treatment time from load of patient data into treatment console to the end of last delivery did not change for the two approaches and was around 13 minutes per patient. These values do not include any imaging or patient positioning procedure — processes that in any case are the same for the two approaches.

**Figure 4:**
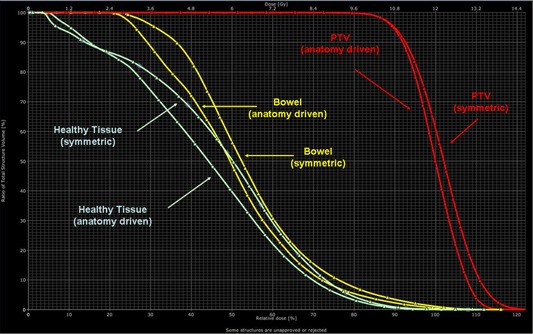
Dose‐volume histograms for the two approaches. The lower dose to healthy tissue and bowel, and the higher dose homogeneity to PTV for the anatomy driven approach, are shown.

Figure 4 shows the DVH for PTV, healthy tissues, and bowels for one patient. Similar results were obtained for the other patients. In particular, the target homogeneity was on average 18% better for the second approach; mean dose to the healthy tissue had a mean reduction of 10% when using the “anatomy driven” approach. Table 1 reports numerical findings from DVH analysis, specifically $D_{50\%}$ and $D_{10\%}$ for PTV and OARs. The data show an overall plan improvement for model (2), though the differences are statistically significant (p<0.05) only for bowel ($D_{50\%}$ and $D_{10\%}$), PTV ($D_{10\%}$) and healthy tissue ($D_{50\%}$). Data in tables are presented as averages over the five investigated patients (errors indicated interpatient variability at one standard deviation level).

**Table 1:** Mean values for the two approaches.

*Structure*	*Model 1*	$D_{50\%}[Gy]$ *Model 2*	*P‐value*	*Model 1*	$D_{10\%}[Gy]$ *Model 2*	*P‐value*
PTV	13.1±0.6	12.9±0.6	>0.05	14.1±0.3	13.3±0.5	<0.05
Bladder	6.4±0.9	6.1±0.7	>0.05	8.2±2.4	8.2±2.4	>0.05
Brain	5.2±0.9	5.1±0.8	>0.05	10.3±1.2	10.1±1.2	>0.05
Bowels	7.3±0.6	6.1±0.4	<**0.05**	11.3±0.9	9.9±0.7	<0.05
Eyes	3.9±0.6	4.0±0.5	>0.05	5.1±0.3	5.0±0.3	>0.05
Healthy Tissue	8.6±0.6	7.7±0.6	<0.05	11.4±1.0	10.8±1.0	>0.05
Heart	5.2±0.5	5.3±0.7	>0.05	8.3±1.0	8.3±0.9	>0.05
Kidneys	5.8±0.7	5.4±0.6	>0.05	8.9±2.2	8.1±1.6	>0.05
Lenses	3.0±0.8	3.1±0.7	>0.05	3.4±0.5	3.4±0.6	>0.05
Liver	6.3±0.3	6.0±0.4	>0.05	10.3±1.6	9.9±1.1	>0.05
Lungs	6.6±0.3	6.4±0.3	>0.05	10.5±1.2	10.3±1.1	>0.05
Oral Cavity	2.3±0.3	2.3±0.3	>0.05	6.7±1.3	6.6±1.5	>0.05
Parotids	4.2±0.7	4.1±0.6	>0.05	5.5±1.2	5.6±0.9	>0.05
Rectum	4.2±0.5	4.0±0.4	>0.05	6.9±1.5	6.8±1.4	>0.05
Spleen	6.2±0.7	6.0±0.5	>0.05	9.9±1.2	9.9±1.2	>0.05
Stomach	4.3±0.4	4.1±0.5	>0.05	9.2±1.2	9.0±1.2	>0.05
Thyroid	2.8±0.1	2.9±0.2	>0.05	6.3±1.0	6.5±1.1	>0.05
Trachea	5.2±0.8	5.1±0.8	>0.05	6.2±0.9	6.3±1.0	>0.05

The normalization method applied ($V_{100\%}=85\%$), which is not fully ICRU compliant but compatible with previous publications, implies that the entire dose distribution to the target is shifted to values higher than the prescription. Nevertheless, RapidArc plans have an average mean dose to PTV lower than 110%, and result in 48% and 39% of the PTV receiving a dose higher than 110% ($V_{110}$) for the “symmetric” and the “anatomy driven” models, respectively. The PTV Homogeneity Index was 41 ±6% and 34±5 for the “symmetric” and the “anatomy driven” models, respectively, with an 18% reduction in the second case.

## IV. DISCUSSION

The present study investigated the feasibility of TMI with volumetric‐modulated arc therapy according to the RapidArc method in terms of in‐silico determination of expected dose distributions.

From a geometrical perspective, rotational techniques seem to be adequate for TMI treatment, as the body has a cylindrical shape and the target has in many parts a cylindrical symmetry. These techniques should, therefore, simplify the demanding task of creating a dosimetric sparing of the organs at risk closed to the target volume. In our previous work, we showed VMAT by RapidArc to be feasible in achieving an adequate PTV coverage with good OARs sparing and only 13 minutes of field on time.^(^ ^5^ ^)^ Four isocenters for eight arcs were optimized simultaneously with at least 2 cm of overlap between two adjacent arcs and collimator rotated to 90° (i.e., MLC motion along cranial–caudal direction). This overlap was applied in order to avoid hot or cold spots around the field junctions as assessed in the work by Fogliata et al.^(^ ^11^ ^)^ The isocenters were equispaced, and field apertures were set the same for all arcs to uniformly cover the entire target length (here defined as “symmetric” model). In the same period, other research groups focused on the possibility to deliver TMI by VMAT. In particular, Aydogan et al.^(^ ^6^ ^)^ of Chicago University used nine arcs on nine isocenters, optimizing three subplans since the treatment plan system used by the authors (PRO II) put a limit on the maximal control points allowed during optimization. A similar strategy was used by Han et al.^(^ ^7^ ^)^ of City of Hope, who employed eight arcs with symmetric jaw apertures, arranging eight isocenters along the patient. They optimized using the PRO II optimizer and had to create subplans, too. The mean OARs received, in all these works, was a dose on average lower than 5 Gy (i.e., < 45% of the prescribed dose).

**Figure 5:**
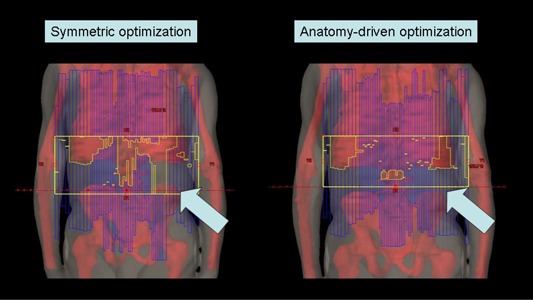
Example of MLC apertures for the two approaches in patient 3: model (1) (left) and model (2) (right). The arrow shows an overexposure of healthy tissues using model (1).

In our previous study, the largest organ exceeding the planning aim to keep $D_{50}$ below 6 Gy was the gastrointestinal cavity. This result is reported also by Han et al.,^(^ ^7^ ^)^ where the authors observed that the maximum increase in $D_{50}$ compared to the HT plans is in the intestines. They speculated as a possible reason of this difference for very large OARs to be identified in the diverse behavior of the MLC systems. In the linac approach, the maximum distance travelled by a leaf between a control point and the following one is limited by the finite speed of the Millennium 120 MLC leaves (in the case of a Varian linac). In HT, instead, the MLC is a binary system (on‐off) in which the leaf position varies instantly. These MLC characteristics affect optimization, especially for large volumes. Hence, taking this into account, we looked for different optimization procedures in order to further reduce dose at the gastrointestinal cavity. The aim was to drive the optimization in order not to excessively stress the MLC motion and avoid priority conflicts in the same field. In the first tests, we tried to use different collimator angles of 90°, but without finding comparable results due to the difficulties to minimize the cost function. The probable reason is the incredible amount of data to manage both in terms of target coverage (around 7000 cc according to our previous work)^(^ ^5^ ^)^ and healthy tissue sparing (i.e., body minus target, around 50000 cc). The best solution to minimize the optimization process could be to “see” each voxel of the CT dataset by only one arc to be unique. Thus, the solution of rotating the collimator to 90° and opening the Y1‐Y2 jaws to the maximum value could be a reasonable choice. Once the collimator setting was defined, we focused on other parameters, looking for solutions that could facilitate the optimization. We visually analyzed each beam aperture and MLC projection. Situations similar to the one reported in Fig. 5(a) suggested to us to try to modify the jaw apertures in order to avoid the occurrence of target in the two edges of the field with healthy tissue in the middle part. In this case, conflicting objectives occur: high dose to the borders but low dose in the center of the jaw apertures along the MLC motion direction. However, the request to overlap consecutive arcs to allow for fields junction and the huge cranial–caudal direction (around 120–130 cm) meant that we had to modify also the isocenter's position. Thus, we tried a second optimization, changing each arc aperture based on the request to minimize or, if possible, delete arcs with target in the edges of the X1‐X2 apertures and healthy tissue in the middle part. This approach plays an important role in particular for the arcs in abdominal region, where most of the caudal ribs and the upper iliac wings are less than 10 cm distant and thus, with very high probability, will be included in the same arc during optimization. The area included into this 10 cm is largely the gastrointestinal track. Thus, the anatomy driven optimization helps to reduce the dose. Furthermore, the reduction of around 7% in MU amount leads to a minor scattered dose. Figure 5 exemplifies, though for an instantaneous MLC and gantry position, the difference between the two approaches. The conflicting objectives pursued in different field zones (e.g., PTV in the field edges and healthy tissue in the middle) lead to an overexposure of healthy tissues for model (1), with its consequent overdosage.

Concerning the choice of isocenter's position and the jaw's aperture, each patient had a specific solution based on anatomy shape. The parameters were decided by the planner according to his personal experience. In this context, the authors want to mention the essential role of selecting the best optimization strategy in order to improve the final plan, as pointed out in recent literature on VMAT techniques.^(^ ^12^ ^–^ ^17^ ^)^

Among the limits of this feasibility study, it is relevant to notice that target definition is, in TMI, extremely complex and labor‐intensive, and requires a dedicated team including radiation oncologists, hematologists, and radiation physicists. All should concur in defining the bones to include in the GTV‐CTV‐PTV, eventually with the aid of special imaging procedures capable to identify the areas of full hematopoietic activity.

As a last remark, it is important to mention that the lower part of the legs is not included in this VMAT study. Similarly, this problem was not addressed in detail in any of the other studies published in literature. Standard approach is to reverse patient position (from head‐first to feet‐first alignment), and to irradiate the lower legs with conventional static fields (i.e., anterior–posterior fields) since no highly sensitive organ at risk is present here. VMAT plans might also be easily generated for the lower legs with one or two arcs and, in this case, an overlapping region with the higher sector should be considered to eliminate risk of cold or hot spots.

Pretreatment QA was not objective of this study, and we referred to our previous study where we demonstrated the deliverability of RA for TMI for the first optimization choice. Similar but lower total MU (thus lower modulation) were required for the anatomy‐driven optimization and thus we can be confident that the cases simulated in this study are deliverable. In any case, for the cases treated in our institute with the second approach we obtained good QA results.

## V. CONCLUSIONS

In conclusion, this study demonstrated the role of isocenter's positions and jaw's apertures based on bone anatomy for the TBI irradiation. The optimal choice of these parameters increases the optimization freedom in shaping an adequate dose distribution. A clinical protocol is starting in our institute and we decided to use the second approach.
